# PERFORATING LICHENOID REACTION TO AMLODIPINE

**DOI:** 10.4103/0019-5154.41659

**Published:** 2008

**Authors:** Chembolli Lakshmi, C R Srinivas, Bindu Ramachandran, Suma B Pillai, V Nirmala

**Affiliations:** *From the Department of Dermatology, PSG Hospitals and PSGIMSR, Peelamedu, Coimbatore - 4, India. E-mail: srini_cr_1955@yahoo.com*; 1 *From the Department of Pathology, PSG Hospitals and PSGIMSR, Peelamedu, Coimbatore - 4, India*

**Fig. 1:**
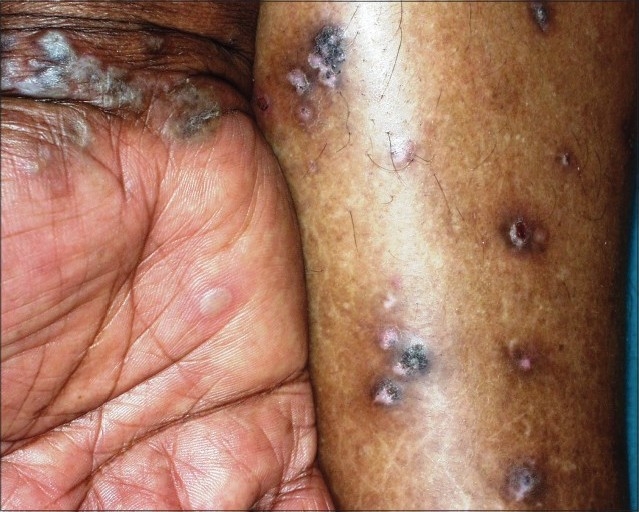
Hyperkeratotic, pigmented, lichenoid papules over the lower limbs

We report here the case of a 54 year-old woman on amlodipine 5 mg for the past six years for systemic hypertension who presented with intensely pruritic, hyperpigmented, keratotic, lichenoid papules topped with white scales over her upper and lower limbs since the last seven months (Fig. 1). The trunk was also involved with less severity. The oral and genital mucosal tissues were normal. She was not on any other medication. Routine investigations including that for blood sugar were within normal limits.

**Fig. 2:**
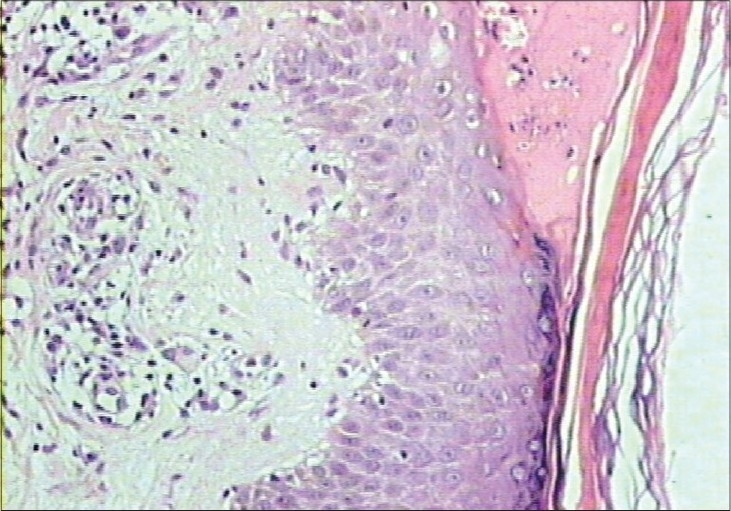
Epidermis shows focal parakeratosis, basal vacuolar damage, lymphocytic exocytosis. Extension of eosinophilic material admixed with polymorphs is seen over the area adjacent to the site of perforation (H and E, ×100)

**Fig. 3:**
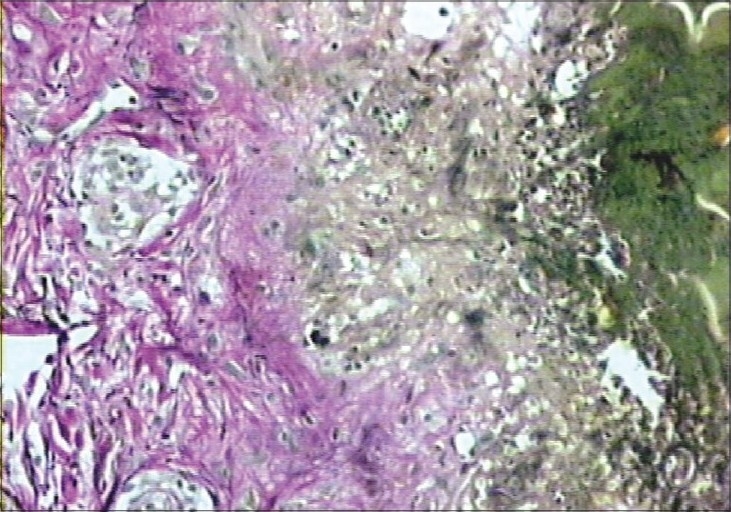
Transepidermal elimination of collagen fibers (Verhoeff van Gieson elastic stained, ×400)

Clinically, transepidermal elimination (TEE) disorder and lichen planus are considered as differential diagnosis. These two conditions were considered as differential diagnostic possibilities. Histological examination showed a lichenoid reaction with transepidermal elimination of collagen (Figs. 2 and 3).

The patient was treated with potent topical corticosteroids, injection triamcinolone acetonide 40 mg/ml IM stat and amlodipine was replaced with losartan 50 mg daily. One month later, all the lesions had subsided leaving postinflammatory hyperpigmentation. The marked symptomatic and clinical improvement following the withdrawal of amlodipine implicates the drug as the most likely cause of the lichenoid papules. Rechallenge with amlodipine was not acceptable to the patient.

Various reactions have been reported with amlodipine including generalized pruritus, erythematous rash, ecchymosis, purpura, urticaria and photosensitivity presenting as telengiectasia.^1^,^2^ Lichenoid reactions may develop after weeks or months following the initiation of therapy.^3^ Although lichen planus has been linked to calcium channel blockers, there are very few reports of amlodipine-associated lichen planus.^4^ Transepidermal elimination with perforation is very rarely seen in classical lichen planus cases.^5^ This finding has not been reported in associaton with lichenoid reactions. A perforating lichenoid reaction could represent a rare, unlisted reaction to amlodipine.
